# Abnormal birth weights for gestational age in relation to maternal characteristics in Sweden: a five year cross-sectional study

**DOI:** 10.1186/s12889-023-15829-y

**Published:** 2023-05-26

**Authors:** Min Wai Lwin, Erika Timby, Anneli Ivarsson, Eva Eurenius, Masoud Vaezghasemi, Sven-Arne Silfverdal, Marie Lindkvist

**Affiliations:** 1 Department of Epidemiology and Global Health, Umeå University, Umeå, Sweden; 2 Department of Clinical Science, Obstetrics and Gynaecology, Umeå University, Umeå, Sweden; 3 Department of Clinical Science, Paediatrics, Umeå University, Umeå, Sweden

**Keywords:** Birth weight, Body mass index, Cross-sectional, Large for gestational age, Lifestyle, Maternal obesity, Small for gestational age

## Abstract

**Background:**

Abnormal birth weight – small for gestational age (SGA) and large for gestational age (LGA) – are important indicators for newborn health. Due to changes in lifestyle in recent decades, it is essential to keep up-to-date with the latest information on maternal factors linked to abnormal birth weight. The aim of this study is to investigate SGA and LGA in relation to maternal individual, lifestyle and socioeconomic characteristics. .

**Methods:**

This is a register-based cross-sectional study. Self-reported data from Sweden’s Salut Programme maternal questionnaires (2010–2014) were linked with records in the Swedish Medical Birth Register (MBR). The analytical sample comprised 5089 singleton live births. A Swedish standard method using ultrasound-based sex-specific reference curves defines the abnormality of birth weight in MBR. Univariable and multivariable logistic regressions were used to examine crude and adjusted associations between abnormal birth weights and maternal individual, lifestyle and socioeconomic characteristics. A sensitivity analysis, using alternative definitions of SGA and LGA under the percentile method, was undertaken.

**Results:**

In multivariable logistic regression, maternal age and parity were associated with LGA (aOR = 1.05, CI = 1.00, 1.09) and (aOR = 1.31, CI = 1.09, 1.58). Maternal overweight and obesity were strongly associated with LGA (aOR = 2.28, CI = 1.47, 3.54) and (aOR = 4.55, CI = 2.85, 7.26), respectively. As parity increased, the odds of delivering SGA babies decreased (aOR = 0.59, CI = 0.42, 0.81) and preterm deliveries were associated with SGA (aOR = 9.46, CI = 5.67, 15.79). The well-known maternal determinants of abnormal birthweight, such as unhealthy lifestyles and poor socioeconomic factors, were not statistically significant in this Swedish setting.

**Conclusions:**

The main findings suggest that multiparity, maternal pre-pregnancy overweight and obesity are strong determinants for LGA babies. Public health interventions should address modifiable risk factors, especially maternal overweight and obesity. These findings suggest that overweight and obesity is an emerging public health threat for newborn health. This might also result in the intergenerational transfer of overweight and obesity. These are important messages for public health policy and decision making.

## Background

Birth weight is a consequence of both foetal and maternal factors and an important indicator of intrauterine growth and newborn health. Abnormal birth weights, such as small for gestational age (SGA) and large for gestational age (LGA), are recognizable risk factors for neonatal morbidity [1, 2]. SGA newborns have subnormal intrauterine growth compared with newborns of the same gestational age and gender, while LGA is a sign of overgrowth [3]. Analysis of maternal causal associations and modifiable risk factors are important from a public health perspective (2). Deviations from expected birth weight have attracted much attention because scientific evidence indicates not only short-term effects, such as neonatal morbidity and mortality, but also adverse long-term effects on subsequent child growth and development, and possibly inter-generational health consequences [4–6].

Epidemiological studies on birth weight have reported that the prevalence of large babies (LGA) has increased over the last few decades in some European countries, including Sweden [5, 7, 8]. More importantly, LGA babies are prone to a condition called macrosomia which is excessive growth beyond a birth weight threshold of 4000 g. Macrosomia is a risk factor for several complications such as shoulder dystocia, Erb’s palsy, fractures of the clavicle or humerus, neonatal asphyxia, hypoglycaemia and hyperbilirubinemia, etc. [9]. Moreover, mothers of these babies are at an increased risk of Caesarean section, obstructed labour, haemorrhage, perineal trauma and maternal complications [9].

SGA babies, on the other hand, have a considerably higher risk of morbidity and mortality in neonatal and post-neonatal periods compared to normal weight babies [1, 10, 11]. The risks are even higher if newborns have both preterm and SGA conditions [10]. These babies have a high risk of several neonatal morbidities such as infections, hypothermia, hypoglycaemia, perinatal respiratory depression, and poor feeding. In addition to these adverse health outcomes, SGA babies can develop childhood growth retardation such as stunting and wasting [6].

Previous newborn studies have shown that birth weight is influenced by maternal health, nutrition, genetics, and environmental and lifestyle factors during pregnancy [12]. Thus, the pregnancy period offers a window of opportunity for health interventions which can potentially benefit both maternal and child health. However, in the Swedish setting, little is known about the prevalence of abnormal birth weight, and associations with pre-pregnancy maternal conditions such as lifestyle and socioeconomic factors.

Lifestyle behaviours have changed considerably in recent decades [13]. Hence there is a need to update knowledge regarding associations between maternal characteristics and newborn weight. For example, one Swedish study in 2013 found that the prevalence of overweight and obesity was high among expectant parents, and suggested that this could be associated with over-nutrition related health problems in newborns [14]. Another Swedish study showed that patterns of high body mass index (BMI) are transferred from parents to toddlers [15], thereby indicating that the intergenerational effects of high BMI deserve more attention. On the other hand, preterm and low birth weight is the most common cause of neonatal mortality in Sweden [16]. Our study will investigate both SGA and LGA as outcome measures to capture birth weight deviations at both ends.

Further knowledge of association between maternal characteristics and abnormal birth weight will inform understanding of intergenerational patterns, and ways of preventing child morbidities and mortalities [17–19]. The aim of this study is to investigate both SGA and LGA in relation to maternal individual, lifestyle and socioeconomic characteristics. Data analysed here cover the maternal pre-pregnancy stage through to childbirth. The study sample includes five years of birth registry data representative of a regional population in Sweden.

## Methods

### Study design and participants

This is a registry based cross-sectional study. Data from the Salut Child Health Promotion Programme in Region Västerbotten, northern Sweden, are enriched with individual level data from the National Board of Health and Welfare [14, 20, 21]. The Salut Programme is a multisector, family centred intervention aimed at reaching all children aged 0–18 and their parents, starting with the parents-to-be before the pregnancy. This has resulted in a prospective longitudinal data collection from 2009 to the present-day. As part of this program, all pregnant women in the Region Västerbotten were provided with a self-reported maternal health and lifestyle questionnaire before their first antenatal care visit. The response rate for the antenatal data collection is around 75%. On average women answer the first antenatal questionnaire at about 11 week gestation.

### Data structure

**Fig. 1:**
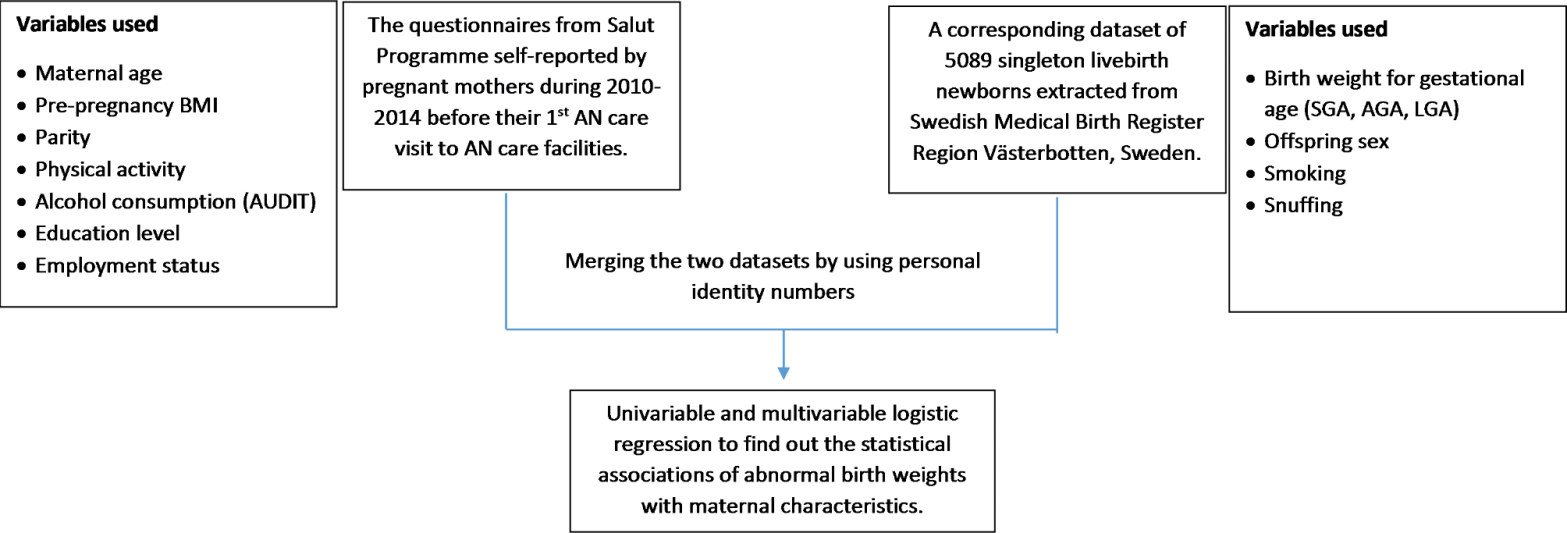
Data source and variable selection for logistic regression analysis. AGA - average for gestational age, AN - Antenatal, AUDIT – Alcohol Use Disorders Identification Test, BMI – body mass index, LGA- large for gestational age, SGA- small for gestational age

The study sample is derived from a merged longitudinal dataset constructed to follow families from pregnancy and through childhood. The Salut antenatal questionnaire which includes important information on lifestyle is enriched by a selection of variables from the MBR hosted by the National Board of Health and Welfare in Sweden. The maternal data from Salut Programme (2010 to 2014) was linked with Swedish Medical Birth Register (using personal identity numbers). A final sample of 5089 singleton newborns and 4683 mothers was obtained for the analysis. Variables selected from the self-reported maternal questionnaire included age at pregnancy, height, pre-pregnancy weight, parity, physical activity, alcohol consumption, employment status and education level. The variables used from the MBR were birth weight, gestational week at birth, maternal smoking and snuffing tobacco at enrolment. See Fig. 1.

The best estimates for dating gestational age at birth were obtained either by the first or second trimester, or the traditional last menstrual period (LMP) method. Gestational dating using ultrasound is offered to all pregnant women in Sweden; about 97% of all pregnant women agree to this. In the final merged dataset, the variables maternal BMI, smoking, and employment had 8.5%, 3.3% and 4.4% of missing data respectively. For the remaining variables missing data was ≤ 2%; maternal age and parity had complete data.

### Dependent variables

Two methods were used to calculate SGA and LGA. They are the percentile method (< 10th & >90th percentile) and the standard deviation method (mean ± 2 Standard deviations) [22, 23]. The second method was used in line with the Swedish MBR. This was calculated according to the ultrasound-based sex-specific Swedish reference curve for normal foetal growth [23]. The percentile methods were used in a sensitivity analysis. Both SGA and LGA for both methods (percentile and standard deviation ) were categorised as binary (SGA or non SGA and LGA or non-LGA).

### Independent variables

#### General characteristics

Pregnant women’s age, height and weight were self-reported in the maternal questionnaires just prior to the first antenatal care visit. Maternal pre-pregnancy BMI was calculated by the formula: BMI = kg/m^2^ = a person’s self-reported weight in kilograms divided by their self-reported height in squared metre. According to WHO’s definition, BMI is classified into underweight (< 18.5 kg/m^2^), normal weight (18.5–24.9 kg/m^2^), overweight (25.0–29.9 kg/m^2^), and obesity (≥ 30 kg/m^2^).

Parity is the number of previous deliveries which is further grouped into primiparity (first pregnancy), multiparity (2nd to 4th pregnancy) and grand multiparity (5th or more). Gestational age at the time of delivery was defined as preterm delivery (< 37 week), term delivery (37–41 week) and post-term delivery (≥ 42week). The offspring’s sex characteristics (male, female) were used in the classification of SGA and LGA, and considered in the data analysis.

#### Lifestyle characteristics

Data about maternal tobacco smoking and snuffing were extracted from the MBR and coded as yes/no questions at the time of enrolment. Data for physical activity and alcohol consumption originated from the Salut maternal questionnaires. This information reflected women’s lifestyle up to 12 months before their pregnancy.

To estimate individual alcohol risk, the Alcohol Use Disorders Identification Test (AUDIT, approved by WHO) was used in the Salut maternal questionnaires to measure the level of alcohol consumption, drinking behaviours and alcohol-related problems within 12 months before the current pregnancy [24]. The AUDIT score ≤ 5 is categorised as non-risky and a score > 5 as risky alcohol consumption. These variables: smoking, snuffing tobacco and alcohol consumption were encoded as binary variables, i.e., having the risk behaviour or not.

The level of physical activity was measured by total duration spent on physical activities during a week. As per WHO recommendation, at least 150 min of moderate-intensity physical activity throughout the week should be maintained for good health in adults, 18–64 years of age, including pregnant women [25]. The level of physical activity was re-coded as hours per week as a continuous variable.

#### Socioeconomic characteristics

Post-secondary education or higher (> 12 years of education) was classified as high education and secondary school level or lower (≤ 12 years of education) as medium or low education. Employment status was categorised as unemployed or employed (either full-time or part-time). These socioeconomic variables were also encoded as binary variables for the analysis.

### Data analysis

Firstly, maternal characteristics and delivery outcomes were summarised in descriptive tables using numbers and proportions. Secondly, birth weights in relation to maternal pre-pregnancy BMI and parity were illustrated by stacked bar graphs. Thirdly, univariable logistic regressions estimated with Generalized Estimating Equations (to handle correlation between siblings) using an exchangeable correlation structure were performed to identify crude associations between SGA and LGA and each maternal characteristic. Lastly, multivariable logistic regression estimated with Generalized Estimating Equations was executed including those variables for which the p-value < 0.05 in the crude analysis. The statistical associations between variables were reported as odds ratios (OR) with 95% confidence intervals (CI). The statistical threshold for level of significance was set to 0.05. All the statistical analyses were performed using Stata16.0 Software.

## Results

In this sample, the mean birth weights of newborns were 3626 g (Standard deviation (SD): 541 g) for males and 3515 g (SD: 506 g) for females. The median gestational age at delivery was 40 weeks (range 25–43) and male-female ratio was 51.2: 48.8. The median birth weight at 40-weeks were 3747 g and 3590 g for male and female newborns. Newborn with macrosomia (> 4000 g) accounted for 18.8% of the sample but low birth weight (< 2500 g) newborns were only 2.8%. The birth weight for gestational age calculations as per Swedish national standard result in 2.8%, 95.6% and 1.6% for LGA, AGA and SGA, respectively.

Around 86.8% of pregnant women were within the age range of 20–35 years. Teenage pregnancies (< 20 years old) were only 0.9% while pregnancies > 35 years of age accounted for 12.4% of the sample. Primiparity contributed to 44.5% of the sample. Almost 30% of women in this local sample were either overweight or obese (19.7% overweight and 9.0% obese), 67.3% of the women had normal BMI and 4% were underweight. Preterm, term and post-term deliveries comprised 4.3%, 88.0% and 7.7%, of the sample respectively.

Regarding maternal lifestyle characteristics, only 1.5% of the women smoked and 3.8% had snuff (tobacco) using behaviour at the enrolment to AN care. According to the AUDIT score, 6.9% of the pregnant mothers had a risk behaviour regarding alcohol consumption during 12 months before the enrolment to ANC. The recommended physical exercise level (150 min/week) was not achieved by 36.1% of the pregnant mothers; 86.2% of the study population were employed and 61.7% had more than 12 years of education. See Table 1.

**Table 1:** Description of the study population

Variables & Categories	n	%
Birth Outcomes		
Birth weight	5089	
> 4000 g	957	18.8%
2500–4000 g	3992	78.4%
< 2500 g	140	2.8%
Size for gestational age	5089	
LGA	141	2.8%
AGA	4865	95.6%
SGA	83	1.6%
Sex of child	5089	
Male	2605	51.2%
Female	2484	48.8%
Maternal general characteristics		
Age at pregnancy	5089	
< 20	44	0.9%
20–35	4416	86.8%
35+	629	12.4%
Parity	5089	
Primiparity	2263	44.5%
Multiparity	2781	54.6%
Grand multiparity	45	0.9%
BMI category	4654	
Underweight (BMI < 18.5)	187	4.0%
Normal weight (BMI 18.5–24.9)	3132	67.3%
Overweight (BMI 25–29.9)	915	19.7%
Obese (BMI ≥ 30)	420	9.0%
Gestational age at delivery	5089	
Preterm (< 37 week)	218	4.3%
Term (37–42 week)	4480	88.0%
Post-term > 42week)	391	7.7%
Lifestyle characteristics		
Smoking at ANC enrolment	5066	
No	4991	98.5%
Yes	75	1.5%
Snuffing tobacco at ANC enrolment	5066	
No	4872	96.2%
Yes	194	3.8%
Alcohol consumption (AUDIT score) within 12 months before pregnancy	5074	
No risk	4722	93.1%
Risky drinker	352	6.9%
Physical activity within 12 months before pregnancy	4985	
Active (≥ 150 min/week)	3185	63.9%
Inactive (< 150 min/week)	1800	36.1%
Socioeconomic characteristics		
Education	4999	
High education > 12 yrs	3083	61.7%
Medium and low education ≤ 12 yrs	1916	38.3%
Employment	4864	
Employed	4192	86.2%
Unemployed	672	13.8%

Note: AUDIT - Alcohol Use Disorders Identification Test, Note: LGA- large for gestational age, AGA - average for gestational age, SGA- small for gestational age

**Fig. 2:**
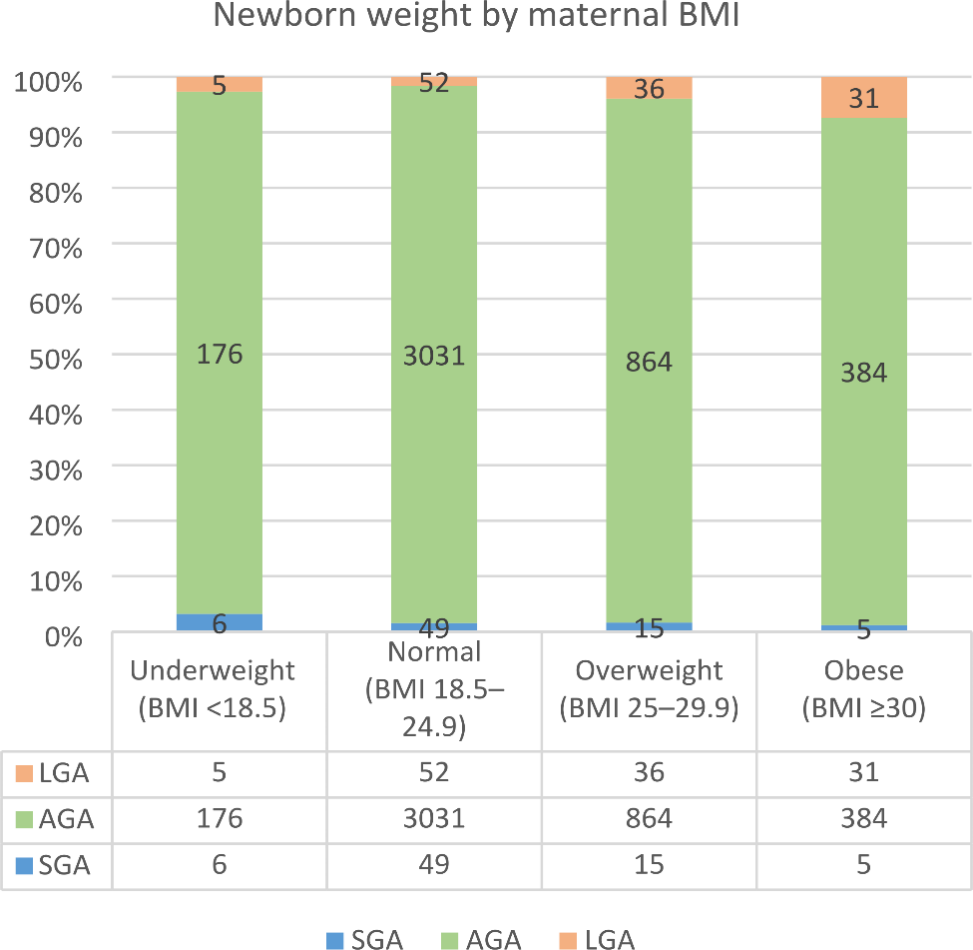
Percentage of birth weight outcomes in different maternal BMI groups. Note: LGA- large for gestational age, AGA - average for gestational age, SGA- small for gestational age

**Fig. 3:**
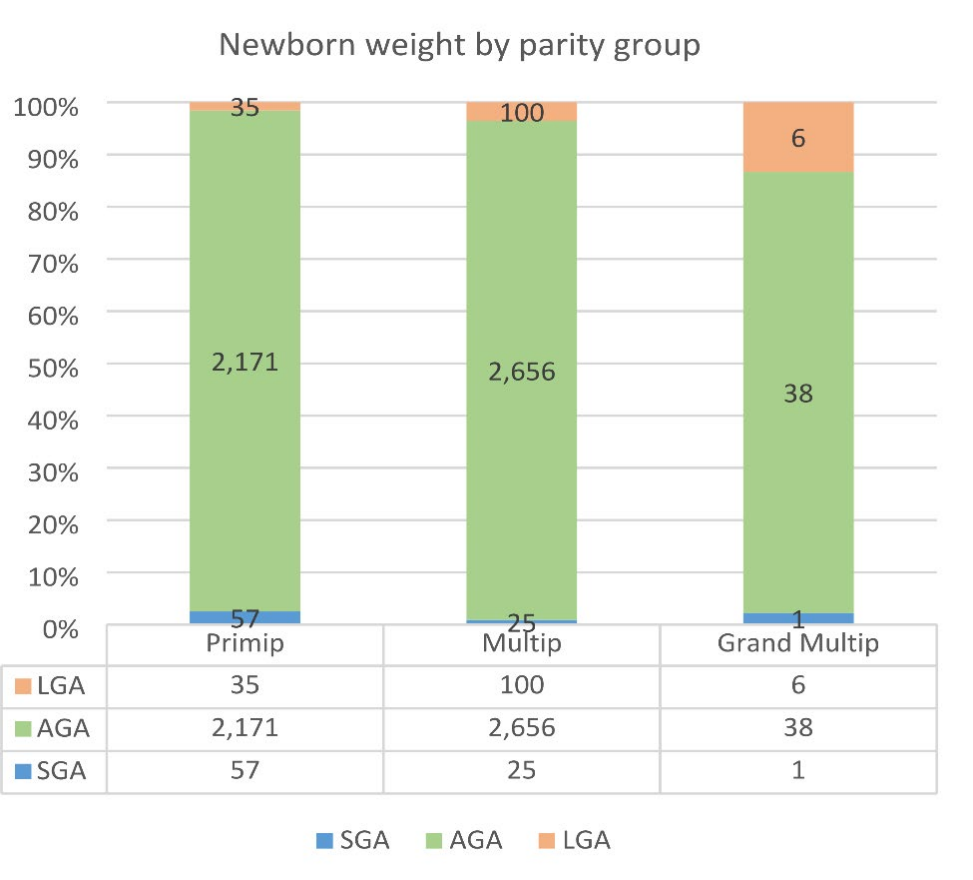
Percentage of birth weight outcome by parity groups. Note: LGA- large for gestational age, AGA - average for gestational age, SGA- small for gestational age, Primip- 1st pregnancy, Multip- 2nd, 3rd or 4th pregnancy, Grand Multip- ≥ 5th pregnancy

The following bar graphs illustrate increasing LGA and decreasing SGA with increasing maternal BMI and parity, as shown in Figs. 2 and 3. Note: missing data may affect the number of observations in each category.

### Result from logistic regression analysis

#### Large for gestational age (LGA)

Older maternal age, parity, high BMI and physical activity showed statistically significant associations with LGA in univariable logistic regression. In the adjusted analysis, a one year increase in maternal age corresponded to an increased odds ratio of 5% for LGA (Adjusted odds ratio (aOR) = 1.05, CI = 1.00, 1.09). The odds for having LGA increased with each pregnancy, (aOR = 1.31, CI = 1.09, 1.58). Overweight and obesity compared with normal BMI group (reference) had significantly higher odds of having LGA, (aOR = 2.28, CI = 1.47, 3.54) and (aOR = 4.55, CI = 2.85, 7.26) respectively. The statistical association with physical activity was attenuated in the adjusted analysis. See Table 2.

#### Small for gestational age (SGA)

The univariable analysis showed that SGA had significant statistical associations with parity and preterm delivery. In the adjusted analysis, those results remained. The odds decreased for each pregnancy (aOR = 0.59, CI = 0.42, 0.81). The association of preterm birth with SGA was found with wide confidence interval (aOR = 9.46, CI = 5.67, 15.79) compared to normal term deliveries. The interpretation of the high odds ratio should be cautious, given the small number of observations with this criterion. See Table 2.

**Table 2:** Univariable and multivariable logistic regression analysis on LGA and SGA.

Independent variables, Maternal factors		Dependent variables, newborn outcome							
		LGA				SGA			
		Crude OR (95% CI)	P value	Adjusted OR (95% CI)	P value	Crude OR (95% CI)	P value	Adjusted OR (95% CI)	P value
**General characteristics**									
**Maternal age**		**1.07 (1.03,1.11)**	< 0.001*	**1.05(1.00,1.09)**	0.032*	0.99(0.95,1.04)	0.729		
**Parity**		**1.49(1.28,1.73)**	< 0.001*	**1.31(1.09,1.58)**	0.005*	**0.57(0.41,0.79)**	0.001*	**0.59(0.42,0.81)**	0.001*
**BMI category**									
	Underweight (BMI < 18.5)	1.63 (0.64,4.16)	0.307	1.83(0.72,4.70)	0.206	2.13 (0.90,5.02)	0.085		
	Normal (BMI 18.5–24.9)	Reference	-	Reference	-	Reference	-		
	Overweight (BMI 25–29.9)	**2.42 (1.57, 3.73)**	< 0.001*	**2.28(1.47,3.54)**	< 0.001*	1.05(0.59,1.89)	0.866		
	Obese (BMI ≥ 30)	**4.73 (2.98,7.50)**	< 0.001*	**4.55(2.85,7.26)**	< 0.001*	0.75(0.30,1.92)	0.552		
**Gestational age at delivery**									
	Preterm (< 37 week)	1.25(0.59,2.64)	0.567			**9.71(5.84,16.15)**	< 0.001*	**9.46(5.67,15.79)**	< 0.001*
	Term (37–41 week)	Reference	-			Reference	-	Reference	-
	Post-term ≥ 42 week)	0.75 (0.37,1.52)	0.421			1.26(0.54,2.95)	0.595	1.10(0.47,2.61)	0.820
**Lifestyle characteristics**									
**Smoking**									
	No	Reference	-			Reference	-		
	Yes	1.54(0.48,4.91)	0.466			1.83(0.44,7.57)	0.402		
**Snuffing tobacco**									
	No	Reference	-			Reference	-		
	Yes	1.05(0.44,2.47)	0.914			0.68(0.17,2.74)	0.588		
**Alcohol consumption**									
	Non-risky drinker	Reference	-			Reference	-		
	Risky drinker	0.52(0.21,1.24)	0.138			0.87(0.35,2.15)	0.767		
**Physical activity, hour per week**		**0.91(0.82,0.10)**	0.049*	0.97(0.87,1.08)	0.53	0.99(0.87,1,12)	0.86		
**Socioeconomic characteristics**									
**Education**									
	High education > 12 years	Reference	-			Reference	-		
	Secondary school level or lower	1.14(0.80,1.61)	0.469			1.19(0.76,1.86)	0.46		
**Employment**									
	Employed	Reference	-			Reference	-		
	Unemployed	1.15(0.72,1.85)	0.553			0.80(0.40,1.61)	0.533		

Note: LGA- large for gestational age, SGA- small for gestational age, * - statistical association is seen

## Sensitivity analysis

The large sample (i.e., 5089 newborns data from regional data collected over 5 years) allowed us to use the percentile method to define SGA and LGA [26]. In this method, SGA and LGA were defined as the newborns weighing < 10th percentile and > 90th percentile of the same gestational week and sex [27, 28]. The calculations were done with a gestational age and weight percentile calculator in Microsoft Excel [29]. This produced the percentile curve of birth weight and cut-off points for respective percentiles of the sample.

For the sensitivity analysis, the logistic regressions were executed with the SGA and LGA determined by the percentile method. The statistical associations with LGA were significant with parity and high BMI, but not with maternal age and physical activity. On the other hand, SGA had statistical association with parity, low BMI, preterm and smoking. Thus, maternal high BMI and parity produced the similar results in the sensitivity analysis. See Tables 3 and 4 for the sensitivity analysis using the WHO percentile method definition of SGA and LGA.

**Table 3:** Birth weight for gestational age classified using the Swedish standard method and the percentile method

Birth weight category	Birth weight classification using Swedish national standards method (as used in Medical Birth Register)		Birth weight classification using WHO percentile method for defining SGA and LGA	
	n	%	n	%
**LGA**	141	2.8%	534	10.5%
**AGA**	4865	95.6%	4029	79.2%
**SGA**	83	1.6%	526	10.3%
**Total**	5089		5089	

**Table 4:** Sensitivity analysis with the SGA and LGA from the percentile method. (Univariable and multivariable logistic regression analysis)

Independent variables, Maternal factors		Dependent variables, newborn outcome							
		LGA				SGA			
		Crude OR (95% CI)	P value	Adjusted OR (95% CI)	P value	Crude OR (95% CI)	P value	Adjusted OR (95% CI)	P value
**General characteristics**									
**Maternal age**		**1.03 (1.01,1.05)**	0.009*	1.01(0.98,1.03)	0.651	**0.97(0.95,0.99)**	0.004*	1.01(0.99,1.04)	0.255
**Parity**		**1.38(1.26,1.50)**	< 0.001*	**1.35(1.21,1.51)**	< 0.001*	**0.57(0.49,0.65)**	< 0.001*	**0.55(0.47,0.64)**	< 0.001*
**BMI category**									
	Underweight (BMI < 18.5)	0.70(0.38,1.31)	0.267	0.73(0.39,1.37)	0.330	**1.81(1.22,2.68)**	0.003*	**1.77(1.18,2.65)**	0.005*
	Normal (BMI 18.5–24.9)	Reference	-	Reference	-	Reference	-		
	Overweight (BMI 25–29.9)	**2.03(1.62,2.53)**	< 0.001*	**1.96(1.57,2.45)**	< 0.001*	**0.75(0.58,0.97)**	0.029*	**0.77(0.59,1.00)**	0.054*
	Obese (BMI ≥ 30)	**2.13(1.59,2.85)**	< 0.001*	**2.08(1.55,2.79)**	< 0.001*	0.84(0.59,1.19)	0.331	0.83(0.58,1.20)	0.326
**Gestational age at delivery**									
	Preterm (< 37 week)	0.86(0.56,1.41)	0.607			**2.53(1.81,3.55)**	< 0.001*	**2.11(1.45,3.08)**	< 0.001*
	Term (37–41 week)	Reference	-			Reference	-	Reference	-
	Post-term ≥ 42 week)	0.87(0.61,1.24)	0.446			0.88(0.61,1.27)	0.498	0.75(0.51,1.11)	0.152
**Lifestyle characteristics**									
**Smoking**									
	No	Reference	-			Reference	-	Reference	-
	Yes	0.77(0.33,1.77)	0.540			**2.21(1.24,3.96)**	0.007*	**2.55(1.36,4.78)**	0.003*
**Snuffing tobacco**									
	No	Reference	-			Reference	-		
	Yes	0.70(0.41,1.20)	0.194			0.88(0.53,1.44)	0.611		
**Alcohol consumption**									
	Non-risky drinker	Reference				Reference	-		
	Risky drinker	0.79(0.54,1.18)				1.31(0.95,1.82)	0.100		
**Physical activity, hour per week**		0.96(0.91,1.00)	0.91			1.04(0.99,1,09)	0.112		
**Socioeconomic characteristics**									
**Education**									
	High education > 12 years	Reference				Reference	-		
	Secondary school level or lower	1.11(0.92,1.34)	0.266			1.07(0.88,1.29)	0.508		
**Employment**									
	Employed	Reference				Reference	-		
	Unemployed	1.11(0.85,1.44)	0.441			1.00(0.77,1.31)	0.464		

Note: LGA- large for gestational age, SGA- small for gestational age, * p-value < 0.05

## Discussion

The main finding was that the occurrence of LGA (2.8%) was more common than SGA (1.6%) in this sample of Swedish newborns. The high prevalence of macrosomia babies (18.8%) compared to the low birth weight (2.8%) further supports this fact. The international standard of newborn weight in the multinational newborn cross-sectional study of the INTERGROWTH21st Project reported the 50th percentile birth weight of newborns at 40-week gestational age as 3380 g for males and 3260 g for females [28]. The corresponding median values of birth weight at 40-weeks in this local sample were 3747 g and 3590 g for male and females respectively. This means that newborn babies in our sample had, on average, a 349 g higher birth weight than the international standard. But there is need for cautious interpretation because the sample in INTERGROWTH study excluded mothers with extreme age, height and BMI.

In high income countries like Sweden, maternal over-nutrition is more common than under-nutrition. This claim is supported by the percentage of maternal BMI categories which are largely occupied by overweight (19.7%), and obesity (9%) compared to the underweight (4%). It is also reasonable that overweight and obese mothers deliver large babies [30]. Overweight and obesity is one of the top uprising public health concerns due to a global increasing trend in mean BMI since 1980 [31, 32]. Associations between toddlers’ and parents’ BMI is shown in another Swedish study [15]. This is indicative that maternal pre-pregnancy overweight and obesity is increasingly becoming a public health problem in maternal and child health in Sweden.

Maternal overweight and obesity are modifiable risk factors and largely preventable with diet, lifestyle, and weight control measures [33]. Thus, there is a window of opportunity in the pre-conception phase for reducing the attributable risk of LGA through protective lifestyle behaviours, such as eating a healthy diet, promoting physical activity, and weight control measures where appropriate. As a prevention strategy of LGA, weight loss and lifestyle interventions should target overweight and obese women during preconception care, or more ideally before childbearing i.e., promoting healthy eating and physical activity during childhood and adolescence. Since some childhood nutritional status originates in the foetal period, this suggests that public health interventions need to focus on the continuum of maternal and child health care, starting at pre-conception.

The Salut program holds a continuous data collection over decades. This is an advantage for monitoring the lifestyle and socioeconomic conditions of parents-to-be in the Swedish population and the relationship with child health. One of the most important findings was that there was no significant statistical association between abnormal birth weight and the well-known maternal determinants of abnormal birthweight such as unhealthy lifestyle habits and poor socioeconomic factors. This negative finding indicates that these factors are less prominent in the Swedish context. This could be a sign of over nutrition which has become a new challenge re-directing some attention from health problems associated with low socioeconomic status.

There is strong scientific evidence that smoking can cause preterm deliveries and decreased birth weight [34–36]. In Sweden daily smoking rates in adults have declined over time according to the latest report from Public Health Agency [37]. The report says 6% of men and women smoked daily in 2021. Snuff use among 16–84-year-old women is estimated at 6% in 2021. In our sample, only a small proportion of respondents (1.5% smoking and 3.8% snuffing) reported these unhealthy lifestyle characteristics. This may explain why no associations between smoking and SGA were found.

In terms of socioeconomic characteristics, abnormal birth weights had no significant statistical association with maternal education level and employment. In general, education is a strong determinant of low-birth-weight outcomes. Most pregnant women in the sample had post-secondary education level or higher (> 12 years of education), and the percentage of women who had less than 9 years of school education in the maternal questionnaire was < 1% (not mentioned in the tables). This might be a reason why education does not determine abnormal birth weight in this Swedish setting.

Indeed, moderate physical exercise is necessary for healthy lifestyle whereas the sedentary lifestyle (low physical activity) is highly associated with overweight and obesity. In our analysis, the physical activity hours per week had a significant association with a decreased LGA in the univariable analysis. Yet the statistical association was diminished in adjusted analysis. This could be due to the strong mediation effect of overweight and obesity in the association pathway. From a public health perspective, unhealthy lifestyles are considered potentially modifiable by means of public health interventions. The health services should continue to provide a good family planning service, adequate preconception care, and proper antenatal care, giving women opportunities to make lifestyle changes, reduce modifiable risk factors and optimise medical conditions [38].

This study further supports relationships between maternal factors and abnormal birth weights. Among the independent variables, the maternal age, parity, pre-pregnancy BMI and preterm deliveries were prominent factors associated with abnormal birth weight in newborns. But recognised health determinants, such as lifestyle and socioeconomic factors, did not have any significant associations with abnormal birth weight in this Swedish population.

## Strengths and limitations

This study used a large sample collected over a 5 year period. A wide range of maternal variables were used for analysing abnormal birth weights on both ends (SGA and LGA). The exposure was measured at the early time of pregnancy which reflects the lifestyle of women in reproductive age in a general population, and the outcomes were measured at the time of delivery. However, only a small proportion of the study participants had an unhealthy lifestyle, i.e., 1.5% smoking, 3.8% snuffing tobacco and 6.9% risky alcohol consumption. Thus, the statistical power for analysis of these specific lifestyle characteristics was low. Further research with larger sample sizes, or focused study designs for specific lifestyles, is needed to increase knowledge about maternal lifestyle and the newborn birth weight deviances.

Since the self-reported questionnaires were used in data collection, there was a slight chance of a recall bias from the respondents, for instance, under-reporting of unhealthy lifestyles and women’s body weight. Such conditions could result in an information bias and might lead to an under-estimation of the associations between maternal characteristics and abnormal birth weights. Moreover, pregnant women often make positive lifestyle changes during pregnancy period which might reduce the risk of abnormal birthweight outcomes.

Maternal morbidity during pregnancy and genetic factors have direct relevance for birth weight in addition to maternal lifestyle, social and environmental factors. For instance, maternal health conditions such as gestational diabetes mellitus, and excessive gestational weight gain could lead to over-nutrition of the foetus resulting in a LGA baby at birth [39]. Small-sized newborns and preterm births could be manifested by maternal complications such as pre-eclampsia, pregnancy hypertension and other medical diseases [40]. However, in this study, such maternal morbidity conditions could not be included in the analysis due to data limitations.

## Conclusion

The main findings in this research suggest that multiparity, maternal pre-pregnancy overweight or obesity are strong determinants for LGA babies. Public health interventions should address modifiable risk factors especially maternal overweight and obesity. For public health policy level decision making, our study highlights that overweight and obesity is a new public health threat affecting newborn health, and that this might also convey an intergenerational transfer of overweight and obesity.

## Data Availability

The datasets presented in this article are not readily available because Region Västerbotten originally collected the data for a child health survey (https://www.regionvasterbotten.se/salut). We accessed data for the present study after approval from both the Region Västerbotten and the Ethical Vetting Board. The data are not publicly available but access for replication analyses is possible. Requests to access the datasets should be directed to (https://www.regionvasterbotten.se/salut) or to marie.lindkvist@umu.se.

## Abbreviations

AGA: Average for gestational age
AUDIT: Alcohol Use Disorders Identification Test
aOR: Adjusted odds ratios
BMI: Body Mass Index
CI 95%: Confidence interval
LGA: Large for gestational age
MBR: Medical Birth Register
SD: Standard deviation
SGA: Small for gestational age
WHO: World Health Organisation
